# Dynamic virulence‐related regions of the plant pathogenic fungus *Verticillium dahliae* display enhanced sequence conservation

**DOI:** 10.1111/mec.15168

**Published:** 2019-07-29

**Authors:** Jasper R. L. Depotter, Xiaoqian Shi‐Kunne, Hélène Missonnier, Tingli Liu, Luigi Faino, Grardy C. M. van den Berg, Thomas A. Wood, Baolong Zhang, Alban Jacques, Michael F. Seidl, Bart P. H. J. Thomma

**Affiliations:** ^1^ Laboratory of Phytopathology Wageningen University & Research Wageningen The Netherlands; ^2^ Department of Crops and Agronomy National Institute of Agricultural Botany Cambridge UK; ^3^ Département des Sciences Agronomiques et Agroalimentaires, Equipe Agrophysiologie et Agromolécules, Institut National Polytechnique de Toulouse – Ecole d'Ingénieurs de Purpan Université de Toulouse Toulouse France; ^4^ Provincial Key Laboratory of Agrobiology Jiangsu Academy of Agricultural Sciences Nanjing China; ^5^ Present address: Botanical Institute and Cluster of Excellence on Plant Sciences (CEPLAS) University of Cologne Cologne Germany; ^6^ Present address: Department of Environmental Biology University La Sapienza Rome Italy; ^7^ Present address: Department of Biology, Theoretical Biology & Bioinformatics Utrecht University CH Utrecht The Netherlands

**Keywords:** comparative genomics, effector, genome evolution, mutagenesis, two‐speed genome, Verticillium wilt

## Abstract

Plant pathogens continuously evolve to evade host immune responses. During host colonization, many fungal pathogens secrete effectors to perturb such responses, but these in turn may become recognized by host immune receptors. To facilitate the evolution of effector repertoires, such as the elimination of recognized effectors, effector genes often reside in genomic regions that display increased plasticity, a phenomenon that is captured in the two‐speed genome hypothesis. The genome of the vascular wilt fungus *Verticillium dahliae* displays regions with extensive presence/absence polymorphisms, so‐called lineage‐specific regions, that are enriched in in planta‐induced putative effector genes. As expected, comparative genomics reveals differential degrees of sequence divergence between lineage‐specific regions and the core genome. Unanticipated, lineage‐specific regions display markedly higher sequence conservation in coding as well as noncoding regions than the core genome. We provide evidence that disqualifies horizontal transfer to explain the observed sequence conservation and conclude that sequence divergence occurs at a slower pace in lineage‐specific regions of the *V. dahliae* genome. We hypothesize that differences in chromatin organisation may explain lower nucleotide substitution rates in the plastic, lineage‐specific regions of *V. dahliae*.

## 1 INTRODUCTION

Microbes colonize near all habitats on earth, even those that are characterized by extreme conditions with respect to salinity, temperature, pH, radiation and pressure (Pikuta, Hoover, & Tang, 2007). How microbes can adapt to their environment, especially when it concerns heterogeneous environments, is a fundamental question in evolutionary biology. It remains a major challenge to determine what the molecular consequences are of adaptive mutations in response to dynamic environments, and how these translate into conditionally adaptive phenotypes. During their life cycle, many microbes colonize other organisms that act as their hosts. These symbiotic interactions between microbes and their hosts can range from commensalistic to either beneficial or parasitic. Plant‐pathogen interactions are often exquisite models for the study of the molecular processes of (microbial) adaptation to fluctuating (host) environments, due to the short generation time of the microbe, the typically well‐characterized interaction in the wild and in the laboratory, and more and more frequently the genetic tractability of the pathogen and the host.

To establish their parasitic relationships, pathogenic microbes evolve repertoires of secreted proteins, so‐called effectors, that mediate host colonization often by deregulating host immunity (Cook, Mesarich, & Thomma, 2015; Dodds & Rathjen, 2010). As plants have evolved immune receptors that recognize various molecular patterns that betray microbial invasion as so‐called invasion patterns, receptors evolved that can detect effectors or their activities (Cook et al., 2015; Dodds & Rathjen, 2010). Consequently, pathogens and their hosts are typically engaged in co‐evolutionary arms races in which plant pathogen effector repertoires are subject to selective forces that often result in rapid diversification. Interestingly, effector genes are often not randomly organized in genomes of filamentous plant pathogens (Dong, Raffaele, & Kamoun, 2015). For instance, effector genes of the oomycete potato late blight pathogen *Phytophthora infestans* reside in repeat‐rich regions that display increased structural polymorphisms and enhanced levels of positive selection (Haas et al., 2009; Raffaele et al., 2010). Consequently, it has been proposed that many filamentous pathogens have a bipartite genome architecture with housekeeping genes residing in a conserved core genome and effector genes in dynamic and repeat‐rich compartments; a “two‐speed” genome (Croll & McDonald, 2012; Raffaele & Kamoun, 2012). It is hypothesized that this compartmentalization facilitates the rapid evolution of effector repertoires to mediate continued symbioses between pathogen and the plant host. Often, repeat‐rich genome regions display signs of such accelerated evolution with structural variations such as presence/absence polymorphisms (Raffaele et al., 2010) or chromosomal rearrangements (Faino et al., 2016; de Jonge et al., 2013). Furthermore, such regions can also display increased substitution rates (Cuomo et al., 2007; van de Wouw et al., 2010), including increased levels of nonsynonymous substitutions (Raffaele et al., 2010; Sperschneider et al., 2015; Stukenbrock et al., 2010).

*Verticillium* is a genus of soil‐borne Ascomycete fungi containing notorious plant pathogens of numerous crops (Inderbitzin & Subbarao, 2014) that infect their hosts via the roots and then colonize xylem vessels, resulting in vascular occlusion and wilt disease (Fradin & Thomma, 2006). Currently, 10 *Verticillium* species are described, which are divided in two phylogenetic clusters, i.e., clade Flavexudans and clade Flavnonexudans (Inderbitzin et al., 2011). *Verticillium* species are thought to have a predominant, if not exclusive, asexual reproduction as a sexual cycle has never been described for any of the species (Short, Gurung, Hu, Inderbitzin, & Subbarao, 2014). Nevertheless, *Verticillium dahliae* still appears to have the machinery for sexual recombination, as mating types and meiosis‐specific genes occur (Short et al., 2014). However, a severely skewed mating type ratio in the global *V. dahliae* population (99% vs. 1%; Short et al., 2014) combined with the low mobility of *V. dahliae* due to its soil‐borne nature makes sexual recombination only a marginal phenomenon at most. In *V. dahliae*, the most notorious plant pathogen within the genus that infects hundreds of plant species (Inderbitzin & Subbarao, 2014), mechanisms different from meiotic recombination were shown to contribute to the genomic diversity, including large‐scale genomic rearrangements, horizontal gene transfer, and transposable element (TE) activity (Faino et al., 2016; de Jonge et al., 2012, 2013; Seidl & Thomma, 2014; Shi‐Kunne, van Kooten, Depotter, Thomma, & Seidl, 2019). These mechanisms often converge on lineage‐specific (LS) regions that are enriched in TEs and in in planta‐induced effector genes (Faino et al., 2016; de Jonge et al., 2013; Klosterman et al., 2011). We previously reported that LS regions of *V. dahliae* are largely derived from segmental duplications (Faino et al., 2016) that are known as important sources for functional diversification (Magadum, Banerjee, Murugan, Gangapur, & Ravikesavan, 2013). To study the evolution of the LS regions, in the present study we exploited comparative genomics across the *Verticillium* genus to identify differential rates of sequence diversification to further characterize the two‐speed genome of *V. dahliae*.

## 2 MATERIALS AND METHODS

### 2.1 Genome sequencing and assembly

Genomes of *Verticillium albo‐atrum* PD747, *Verticillium alfalfae* PD683, *V. dahliae* JR2 and VdLs17, *Verticillium isaacii* PD618, *Verticillium klebahnii* PD401, *Verticillium nubilum* PD621, *Verticillium tricorpus* PD593 and MUCL9792, *Verticillium zaregamsianum* PD739 were previously assembled (Faino et al., 2015; Klosterman et al., 2011; Seidl et al., 2015; Shi‐Kunne, Faino, Berg, Thomma, & Seidl, 2018) and sequence reads of *Verticillium nonalfalfae* isolates TAB2 and Rec are publicly available (Bioproject PRJNA283258; Jelen, Jonge, Peer, Javornik, & Jakse, 2016). *Verticillium* strains CQ2, 85S, PD670, PD660, PD659 and PD736 were newly sequenced. To this end, we isolated genomic DNA from potato dextrose broth cultures as previously described (Seidl et al., 2015). *Verticillium dahliae* strains CQ2 and 85S were sequenced on the PacBio RSII platform (Pacific Biosciences of California, CA, USA; Faino et al., 2015). Briefly, DNA was mechanically sheared and size selected using the BluePippin preparation system (Sage Science, Beverly, MA, USA) to produce ~20 kb size libraries. The sheared DNA and final library were characterized for size distribution using an Agilent Bioanalyzer 2100 (Agilent Technology, Inc., Santa Clara, CA, USA). The PacBio libraries were sequenced on four SMRT cells per *V. dahliae* isolate on a PacBio RS II instrument using the P6–C4 polymerase‐chemistry combination and a >4 hr movie time and stage start. Filtered subreads for CQ2 and 85S, were assembled using the HGAP version 3 protocol (Table S1; Chin et al., 2013).

For PD670, PD660, PD659 and PD736, two libraries (500 bp and 5 kb insert size) were prepared and sequenced using an Illumina High‐throughput sequencing platform. In total, ~18 million paired‐end reads (150 bp read length; 500 bp insert size library) and ~16 million mate‐paired read (150 bp read length; 5 kb insert size library) were produced per strain. We assembled the genomes using the A5 pipeline (default settings; Tritt, Eisen, Facciotti, & Darling, 2012), and we subsequently filled the remaining sequence gaps using SOAPdenovo2 (default settings; Luo et al., 2012). After obtaining final assemblies, we used quast (Gurevich, Saveliev, Vyahhi, & Tesler, 2013) to calculate genome statistics. Gene annotations for *V. dahliae* strain JR2 and other *Verticillium* species were obtained from previous studies (Faino et al., 2016; Shi‐Kunne et al., 2018), except for *V. isaacii* strain PD660 that was annotated with the Maker2 pipeline in this study according to Shi‐Kunne et al. (2018) and Holt and Yandell (2011).

### 2.2 Comparative genome analysis

Repetitive elements were identified using repeatmodeler (version 1.0.8) based on known repetitive elements and on de novo repeat identification, and genomes were subsequently masked using repeatmasker (version 4.0.6; sensitive mode; Smit, Hubley, & Green, 2015). To prevent assigning high sequence identities to repetitive elements, sequence alignments were performed to a repeat‐masked reference genome using nucmer (option ‐‐maxmatch), which is part of the mummer package (version 3.1; Kurtz et al., 2004).

Linear plots showing alignments within and closely adjacent JR2 LS regions were plotted with the r package genoPlotR (version 0.8.7; Figure 2; Guy, Kultima, & Andersson, 2011). Alignments >7.5 kb in length were depicted along the reference genome with the r package Rcircos (version 1.2.0; Figure 3; Zhang, Meltzer, & Davis, 2013). Lineage‐specific sequences were defined by alignment of different strains to a reference using nucmer (version 3.1, option ‐‐maxmatch; Kurtz et al., 2004) and regions were determined using bedtools version 2.25.0 (options sort, merge and genomecov; Quinlan & Hall, 2010).

Lineage‐specific regions of *V. dahliae* and *V. tricorpus* were delimited based on interspecific nucleotide alignments with nucmer, which is part of the mummer package (version 3.1, option ‐‐maxmatch; Kurtz et al., 2004). Genome regions were high sequence conservations started or ended in combination with absence/presence polymorphisms were determined as start or end of the LS region, respectively (Table S6). The pairwise identity of the genome‐wide and LS regions between *V. dahliae*/*V. tricorpus* and other haploid *Verticillium* species was calculated using nucmer (option ‐‐maxmatch) by dividing the respective *V. dahliae/V. tricorpus* query sequences into nonoverlapping windows of 500 bp and align them to the repeat‐masked genomes of *Verticillium* species (Table 1). Only sequences with a 1‐to‐1 alignments and with a minimum alignment length of 500 were considered (delta‐filter −1 –l 500). In this fashion, genome‐wide sequence identity between *Verticillium* species was also calculated to determine the sequence divergence across the *Verticillium* phylogenetic tree (Figure 4). Instead of using *V. tricorpus* and *V. dahliae* as reference genomes, *V. klebahnii* and *V. alfalfae* were used as reference for clade Flavexudans and clade Flavnonexudans respectively, to calculate the genome‐wide nucleotide identity differences with the remaining species within the same clade. Nucleotide identity differences with remaining species were calculated in increasing order of phylogenetic distance with the reference species. Identity differences were evenly assigned to the phylogenetic branches in increasing order of phylogenetic distance.

**Table 1:** Sequence identities between *V. dahliae* and *V. tricorpus* with other haploid *Verticillium* species (excluding repetitive regions)

	Species/strain	Core‐genome (%)		LS regions (%)		Number of windows aligned to LS regions^a^	*p*‐Value identity^b^	*p*‐Value variance^c^
		Md	VAR	Md	VAR			
*Verticillium dahliae*	*V. albo‐atrum*	88.0	14.1	97.6	14.2	153	<2.2e−16	.0028
	*V. alfalfae*	94.2	15.2	100.0	12.5	454	<2.2e−16	<2.2e−16
	*V. nonalfalfae*	94.4	15.5	100.0	7.3	1,018	<2.2e−16	<2.2e−16
	*V. nubilum*	88.4	11.5	95.2	30.8	98	<2.2e−16	2.65 e−05
	*V. tricorpus*	88.2	12.4	97.6	13.8	153	<2.2e−16	.063
	*V. isaacii*	88.4	12.5	98.6	22.5	170	<2.2e−16	.0067
	*V. klebahnii*	88.4	12.6	97.9	23.5	82	<2.2e−16	.080
	*V. zaregamsianum*	88.8	13.5	92.5	17.0	31	2.12e−06	.51
*Verticillium tricorpus*	*V. albo‐atrum*	88.9	16.1	100	11.3	609	<2.2e−16	<2.2e−16
	*V. alfalfae*	88.2	16.0	90.9	NA	1	0.38	NA
	*V. dahliae*	88.5	14.5	97.8	11.1	161	<2.2e−16	.00021
	*V. nonalfalfae*	88.2	15.3	99.4	30.4	26	3.0e−12	.018
	*V. nubilum*	88.2	14.4	95.4	11.9	74	<2.2e−16	.39
	*V. isaacii*	94.6	16.2	98.7	23.4	146	<2.2e−16	.39
	*V. klebahnii*	94.8	16.1	98.4	15.3	189	<2.2e−16	.036
	*V. zaregamsianum*	94.8	15.7	92.4	12.3	47	0.0018	.40

Note

The strains used in this table: *V. albo‐atrum*, PD747; *V. alfalfae*, PD683; *V. dahliae*, JR2; *V. nonalfalfae*, TAB2; *V. nubilum*, PD621; *V. tricorpus*, PD593; *V. isaacii*, PD660; *V. klebahni*, PD401; *V. zaregamsianum*, PD739.

Abbreviations: Md, median sequence identity; VAR, sequence variation.

a Windows of 500 bp.

b The *p*‐value was calculated with a two‐sided Wilcoxon rank‐sum test.

c The *p*‐value was calculated with the Fligner–Killeen test of homogeneity of variances.

A pan‐LS‐genome was constructed based on following *Verticillium* isolates: JR2 (*V. dahliae*), PD683 (*V. alfalfae*), PD593 (*V. tricorpus*) and PD401 (*V. klebahnii*). Genome regions of these four species with increased sequence conservation were combined (Table S6). Repeat masked regions were removed from the pan‐LS‐genome using bedtools version 2.25.0 (Quinlan & Hall, 2010). Additionally, duplicated regions (≥90% identity, ≥100 bp) in the pan‐LS‐genome were determined using nucmer (version 3.1, option ‐‐maxmatch; Kurtz et al., 2004) and subsequently removed with using bedtools version 2.25.0 (options merge, subtract and getfasta; Quinlan & Hall, 2010). The fraction of pan‐LS‐genome that is present in every individual *Verticillium* strain was determined using nucmer (version 3.1, option ‐‐maxmatch; Kurtz et al., 2004). The clade pan‐LS‐genomes were constructed by combining all the pan‐LS‐genome regions that are present in the *Verticillium* clade isolates, which was then also removed from duplicate regions.

To compare the rate of synonymous and nonsynonymous substitutions between the core and LS regions, *Ka* and *Ks* of orthologs of JR2 and TAB2 were determined using the Nei and Gojobori method (Nei & Gojobori, 1986) in paml (version 4.8; Yang, 2007). Significance of positive selection was tested using a *Z* test (Stukenbrock & Dutheil, 2012). *Z*‐values > 1.65 were considered significant with *p* < .05. Secreted proteins were predicted by SignalP4 (Petersen, Brunak, Von Heijne, & Nielsen, 2011). To compare coding regions of genes and intergenic regions, sequence identities were retrieved by blast (version 2.2.31+) searches between strains *V. dahliae* JR2 and *V. nonalfalfae* TAB2 (Altschul, Gish, Miller, Myers, & Lipman, 1990). The coding regions of genes were aligned to each other and the best hits (sequence identity) with a minimal coverage of 80% with each other were selected. Intergenic regions of *V. dahliae* strain JR2 were fractioned in 5 kb windows with bedtools version 2.25.0 (options makewindows and getfasta) and similarly queried to the genome of *V. nonalfalfae* strain TAB2 (Quinlan & Hall, 2010). Hits with a maximal bit‐score and minimal alignment of 500 bp to a window were selected.

### 2.3 Tree building and ortholog analysis

The phylogenetic trees of the *Verticillium* genus were previously generated using 5,228 single‐copy orthologs that are conserved among all of the genomes (Shi‐Kunne et al., 2018). The phylogenetic tree of *V. dahliae* strains JR2, CQ2, VdLs17 and 85S with *V. alfalfae* as outgroup was constructed using realphy (default settings; Bertels, Silander, Pachkov, Rainey, & Van Nimwegen, 2014).

Orthologous groups of *Verticillium* species were determined by using orthofinder (version 2.2.7, option ‐S diamond; Emms & Kelly, 2015), including *Sodiomyces alkalinus* as outgroup (Grum‐Grzhimaylo et al., 2018). Orthologous groups that are shared among at least three species were selected for phylogenetic tree construction. Individual ortholog groups were aligned using mafft (LINSi; version 7.04b; Katoh, Misawa, Kuma, & Miyata, 2002; Katoh & Standley, 2013). A maximum likelihood phylogeny was inferred using raxml (version 8.2.4) with the GAMMA model of rate heterogeneity and the General Time Reversible (GTR) model of nucleotide substitutions (Stamatakis, 2014). The robustness of the inferred phylogeny was assessed by 100 rapid bootstrap approximations.

## 3 RESULTS

### 3.1 LS sequences reside in four regions of the genome of *V. dahliae* strain JR2

**Figure 1:**
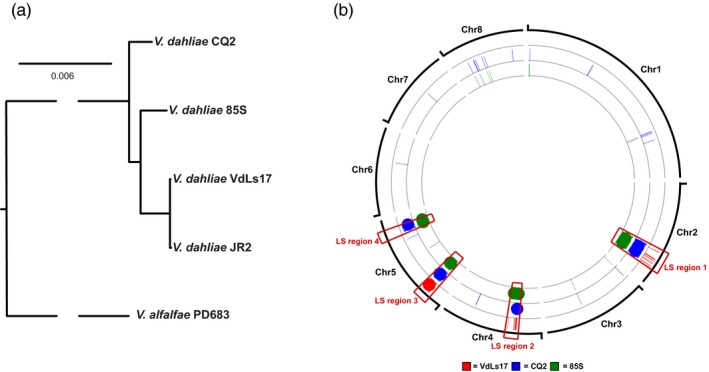
Locations of lineage‐specific (LS) regions in the genome of *Verticillium dahliae* strain JR2. (a) The phylogenetic relationship between *V. dahliae* strains JR2, VdLs17, CQ2 and 85S is shown. The phylogenetic relationship of all *V. dahliae* strains was inferred by single‐copy orthologs. *V. alfalfae* was used as out‐group species. (b) LS regions of *V. dahliae* strain JR2 were determined by individual comparisons to *V. dahliae* strains VdLs17 (red), CQ2 (blue) and 85S (green). Sequences of minimum 7.5 kb without alignment to at least one of the other isolates are depicted in colour at their respective position on the *V. dahliae* strain JR2 genome [Colour figure can be viewed at wileyonlinelibrary.com]

Previously, four LS regions were characterized for *V. dahliae* strain JR2; one on chromosome 2 and 4, and two on chromosome 5 (Faino et al., 2015, 2016; de Jonge et al., 2013). By definition, LS regions in *V. dahliae* isolate JR2 are enriched for sequences that lack synteny to various other *V. dahliae* strains, including the completely sequenced genome of strain VdLs17 (Faino et al., 2015, 2016; de Jonge et al., 2013). Since *V. dahliae* strains JR2 and VdLs17 only recently diverged (de Jonge et al., 2013), we sequenced two additional *V. dahliae* strains that diverged earlier from JR2 (Figure 1a), namely strains CQ2 and 85S that were isolated from cotton in China and sunflower in France, respectively, resulting in assemblies of 35.8 and 35.9 Mb in 17 and 40 contigs, respectively (Table S1). Subsequent alignment revealed that the JR2 genome was not covered by 2.0%, 7.1%, and 6.6% of sequences from VdLs17, CQ2 and 85S, respectively, while 1.4% of the JR2 genome sequence could not be identified in any of the three other *V. dahliae* strains. The vast majority of the JR2 sequences without alignment (88%, 82%, and 91% for VdLs17, CQ2 and 85S, respectively) localize in the four previously identified LS regions that collectively contain 476 predicted protein‐coding genes (Figure 1b). Thus, despite the addition of more diverged *V. dahliae* strains, intraspecific presence/absence polymorphisms keep converging on the four previously identified genomic regions that are thus significantly more dynamic than other parts of the genome.

### 3.2 LS regions share increased sequence identity to other *Verticillium* species

**Figure 2:**
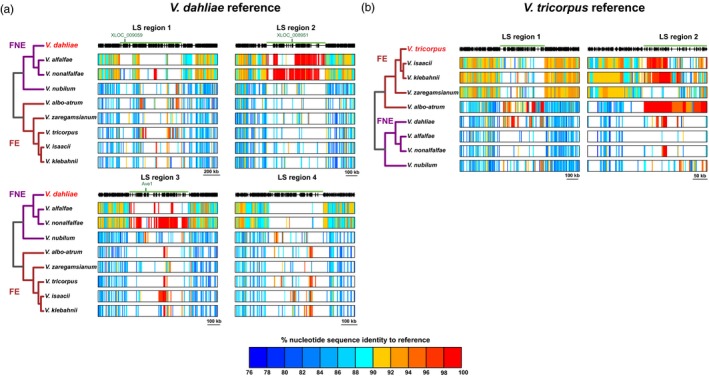
Interspecific alignments and sequence identity within and immediately adjacent to regions with high interspecific sequence identity. The green line indicates regions with high sequence identity. Coloured blocks are corresponding sequences to (a) *Verticillium dahliae* and (b) *V. tricorpus* with the colour indicating the sequence identity. The black, vertical stripes represent gene positions of the reference strains. Locations of characterized *V. dahliae* effector genes are indicated: *Ave1*, *XLOC_008951* and *XLOC_009059* (de Jonge et al., 2012, 2013). Strains used in this figure are *V. tricorpus* PD593, *V. isaacii* PD660, *V. klebahnii* PD401, *V. zaregamsianum* PD739, *V. albo‐atrum* PD747, *V. dahliae* JR2, *V. alfalfae* PD683, *V. nonalfalfae* TAB2 and *V. nubilum* PD621 [Colour figure can be viewed at wileyonlinelibrary.com]

Next, we extended our analysis to other *Verticillium* species. While most of the *V. dahliae* strain JR2 genome aligns with *V. nonalfalfae* strain TAB2 with an average sequence identity of ~92%, particular regions display increased sequence identity, even up to 100% (Figure S1). Intriguingly, the regions with increased sequence identity co‐localize with LS regions (Faino et al., 2015, 2016). To perform interspecific comparisons with all other haploid *Verticillium* species, genomic sequences of *V. dahliae* were aligned in windows of 500 bp to the other species, excluding repetitive regions, displaying median identities ranging from 88% to 94%. These identities correspond to the phylogenetic distances to *V. dahliae* (Table 1). Sequence identities were similarly calculated in windows for the LS regions. Intriguingly, the LS regions displayed significantly increased sequence identities when compared with the core genome, ranging from 92.5% median sequence identity for *V. zaregamsianum*, one of the phylogenetically most distantly related species, to 100% for the most closely related species *V. alfalfae* and *V. nonalfalfae* (Figure 2a, Table 1).

**Figure 3:**
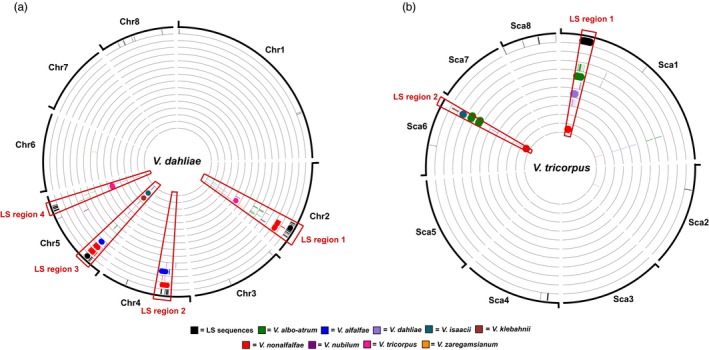
Regions with particularly high interspecific sequence identity. All *Verticillium* strains mentioned in Table S2 were used. (a) Black bars correspond to lineage‐specific (LS) sequences of *V. dahliae* strain JR2 (for details, see Figure 1b). Sequences (≥7.5 kb) with particularly high sequence identity in any of the other *Verticillium* species (≥96% for *V. alfalfae* and *V. nonalfalfae*, ≥90% for all other *Verticillium* species) are plotted at the corresponding position on the genome of *V. dahliae* strain JR2. (b) The eight biggest scaffolds of *V. tricorpus* strain PD593 are depicted as these comprise over 99.5% of the genome. Black bars correspond to LS sequences (≥7.5 kb) in the *V. tricorpus* strain PD593 genome without alignments to *V. tricorpus* strain MUCL9792. Sequences (≥7.5 kb) with particularly high sequence identity in any of the other *Verticillium* species (≥96% *V. isaacii, V. klebahnii* and *V. zaregamsianum*, ≥90% for all other *Verticillium* species) are plotted at the corresponding position on the genome of *V. tricorpus* strain PD593. Nondepicted *Verticillium* strains did not have sequences (≥7.5 kb) with previously mentioned degrees of identity to *V. tricorpus* strain PD593 [Colour figure can be viewed at wileyonlinelibrary.com]

To assess whether high interspecific sequence identity concerns only LS regions, we aligned *Verticillium* sequences to the complete *V. dahliae* JR2 genome. For several species we used multiple strains at this stage (Table S2). Nearly all (99%–100%) of the *V. alfalfae* and *V. nonalfalfae* sequences that display >96% identity to *V. dahliae* strain JR2 sequences localized in LS regions (Figure 3a). Similarly, sequences of at least 100 kb with >90% identity of other *Verticillium* species mapped to *V. dahliae* strain JR2 LS regions, ranging from 70% of the sequences in *V. nubilum* PD621 to 95% in *V. albo‐atrum* PD670 and *V. tricorpus* PD593 (Table S3). Thus, high interspecific sequence identity is specifically associated with LS regions of *V. dahliae*.

### 3.3 High interspecific sequence identity of LS regions is not unique to *V. dahliae*

To investigate whether other *Verticillium* species similarly carry LS regions that display high interspecific sequence identity, we performed alignments using *V. tricorpus* strain PD593 as a reference because of its high degree of completeness with seven of the nine scaffolds probably representing complete chromosomes (Table S2; Shi‐Kunne et al., 2018). Furthermore, this species belongs to the Flavexudans clade, in contrast to *V. dahliae* that belongs to Flavnonexudans. LS sequences of *V. tricorpus* strain PD593 were determined by comparison to *V. tricorpus* strain MUCL9792 (Seidl et al., 2015). In total, 98% of the PD593 genome could be aligned to MUCL9792. However, 48% of the sequences that are specific for *V. tricorpus* strain PD593 reside in only a single genomic region of 41 kb on scaffold 1 (Figure 3b). Like for *V. dahliae* strain JR2, sequences of other *Verticillium* species aligned with high identity to *V. tricorpus* PD593: *V. isaacii*, *V. klebahnii* and *V. zaregamsianum* display a median genome identity of ~95%, while other haploid *Verticillium* species display ~88%–89% median genome identity (Table 1). Notably, regions that display significantly higher sequence identity localized at the LS region on scaffold 1, but also to an additional region of 23 kb on scaffold 6 (Figures 2b and 3b). It is likely this concerns an LS region that could not be identified based on the two *V. tricorpus* strains used in our analysis, and thus will also be referred to as LS region. For *Verticillium* strains with total alignments of at least 100 kb of high‐identity sequences, the fraction of high‐identity sequences that aligned to LS genome regions ranged from 49% for *V. nubilum* (PD621) up to 84% for *V. albo‐atrum* (PD747; Table S4). As expected, the sequence identity to six of the eight other haploid *Verticillium* species was significantly higher in LS genome regions compared to the core genome (Table 1). No increase in sequence identity was found in alignments with *V. alfalfae* strain PD683 and *V. zaregamsianum* strain PD739 as only few sequences of LS genome regions could be aligned (Table 1).

### 3.4 LS regions are unlikely to originate from horizontal DNA transfers among *Verticillium* species

**Figure 4:**
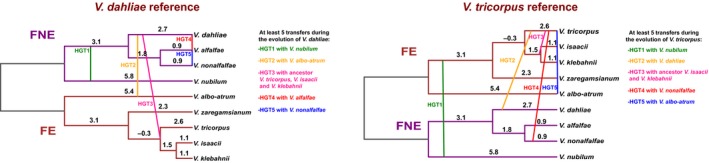
A hypothetical model to explain high levels of sequence identity of LS regions through horizontal transfers between *Verticillium* species. The figure displays the minimum amount of horizontal DNA transfer events that would have been required to shape the LS regions of *V. dahliae* (left) and *V. tricorpus* (right). Putative horizontal transfers are determined based on the difference in median sequence identity between LS and core genomic regions (Table 1). The numbers on the branches display the genome‐wide sequence divergence (in %) between species over the evolutionary time that they were separated. To this end, *V. klebahnii* and *V. alfalfae* were used as reference for clade Flavoxedans and clade Flavnonexudans respectively, to calculate the genome‐wide nucleotide identity differences with the other species within the respective clade. Nucleotide identity differences with other species were calculated in increasing order of phylogenetic distance with the reference species. Identity differences were evenly assigned to the phylogenetic branches in increasing order of phylogenetic distance [Colour figure can be viewed at wileyonlinelibrary.com]

High interspecific sequence identity of particular genomic regions, such as observed here for LS regions, could occur through two different mechanisms: (a) horizontal transfer of sequences between species, in this case horizontal transfer of LS sequences between *Verticillium* species, or (b) differences in nucleotide substitution rates between genomic regions, in this case between core and LS genome regions. To explain the high sequence identity through the occurrence of interspecific horizontal DNA transfers, a minimum of five such transfers must have occurred that involve *V. dahliae* to explain the differences in median sequence identities of LS and core genome regions with the other *Verticillium* species (Figure 4, Table 1). Similarly, at least five such transfers are required to explain the composition of the *V. tricorpus* genome relative to its sister species (Figure 4, Table 1). Consequently, dozens of horizontal DNA transfers among sister species are required to explain the composition of the LS regions throughout the species of the whole *Verticillium* genus.

If LS regions were transferred between species through a limited number of events, a depletion of interspecific sequence identity variation in LS regions may be observed. Thus, we calculated the variance in interspecific sequence identity for *V. dahliae* LS regions and genome‐wide (Table 1). Sequence identities in LS regions varied less than in the core genome in alignments to *V. alfalfae* and to *V. nonalfalfae*, two species that diverged only recently from *V. dahliae* (Table 1). In contrast, sequence identities with *V. albo‐atrum*, *V. nubilum* and *V. isaacii* displayed more variation in LS genome regions than within the core genome regions (Table 1). No significant differences between LS and core genome regions were found in alignments with *V. tricorpus*, *V. klebahnii* and *V. zaregamsianum*. A similar pattern is observed for *V. tricorpus* as LS regions display lower sequence identity variation than the core genome in alignments to *V. albo‐atrum* and *V. dahliae*, whereas the opposite is true for alignments to *V. nonalfalfae* and *V. klebahnii* (Table 1). Thus, although it needs to be noted that there is a significant difference in size between core and LS genomic regions, there seems to be no genus‐wide trend towards lower interspecific sequence identity variation in LS regions compared to the core genome, which may be interpreted as an argument against horizontal transfer of these regions.

**Figure 5:**
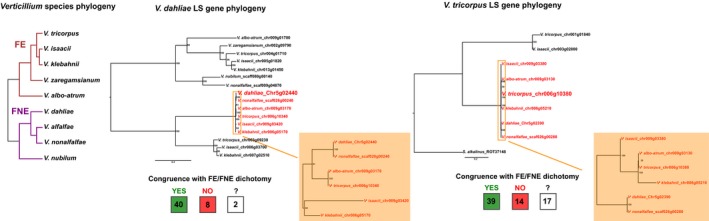
The evolution of lineage‐specific (LS) region genes. The left tree shows the phylogenetic relationships between the haploid *Verticillium* species and their division into clade Flavexudans (FE) and clade Flavnonexudans (FNE). The middle and right phylogenetic trees are of a particular LS region gene from *V. dahliae* and from *V. tricorpus*, respectively. Genes indicated in red have particularly high sequence identity. The robustness of the phylogeny was assessed using 100 bootstrap replicates. For all *V. dahliae* and *V. tricorpus* LS region genes with at least one homolog in clade Flavexudans and one homolog in clade Flavnonexudans, phylogenetic trees were constructed including all their *Verticillium* homologs. The number of trees in congruence and incongruent with the *Verticillium* clade dichotomy are indicated in the green and red box, respectively. In these assessments, complete trees were analysed in case the tree did not contain paralogs for any of the *Verticillium* species. In case of paralogs that indicate gene duplication events, only a subclade of the phylogenetic tree was used for assessment. The number in the white box is the number of trees that was inconclusive [Colour figure can be viewed at wileyonlinelibrary.com]

In case of horizontal transfers, phylogenetic trees of LS genes and their orthologs in other *Verticillium* species are likely to display incongruences with the general *Verticillium* phylogeny. Genes in LS regions often have only few orthologs due to the abundant presence/absence polymorphisms that characterize these regions but, nonetheless, 50 genes could be identified that have at least one ortholog in clade Flavexudans and one in clade Flavnonexudans. To identify (in)congruences with the *Verticillium* phylogeny, we assessed phylogenetic trees for these genes. Due to the generally low resolution that can be attributed to the limited amount of orthologs and their generally high degree of sequence conservation, we focussed our analysis on the dichotomy of homologs from Flavexudans and Flavnonexudans species. For example, Chr5g02240 has homologs in seven other *Verticillium* species, with five highly conserved orthologs in species that generally differ from 5.2% to 11.2% in genome‐wide nucleotide identity with *V. dahliae*, whereas sequence identities between the highly conserved Chr5g02240 orthologs differs only from 0% to 0.8% (2,460 total nucleotides; Figure 5). Moreover, in accordance with the overall *Verticillium* phylogeny, the *V. nonalfalfae* homolog clusters with that of *V. dahliae*. In total, 80% of the trees displayed a phylogeny where the Flavexudans and Flavnonexudans species clustered, whereas only 5% of the trees did not yield this typical dichotomy and 15% of the trees did not contain sufficient homologous sequences to be conclusive (Figure 5). Similarly, the majority (56%) of phylogenetic trees for *V. tricorpus* LS genes obey to the Flavexudans and Flavnonexudans dichotomy (Figure 5). Like for *V. dahliae* gene Chr5g02240, the phylogenetic tree for homologs of *V. tricorpus* gene Chr006g10380 shows the expected segregation of Flavnonexudans homologs from the Flavexudans homologs. Thus, the majority of LS genes can be demonstrated to diverge in accordance with the *Verticillium* clade dichotomy.

Taken together, neither the comparison of interspecific sequence identity variation within and outside LS regions, nor the analysis of phylogenetic trees of LS genes, seems to support a scenario where horizontal DNA transfer events was fundamental to the evolution of LS regions. Moreover, as dozens of such events would be required among the various *Verticillium* species, many of which over a short evolutionary time scale, and considering the low‐mobile, soil‐borne nature of the various species, we deem it very unlikely that interspecific horizontal DNA transfers shaped LS regions.

### 3.5 Pan‐LS‐genome distribution across the *Verticillium* genus

**Figure 6:**
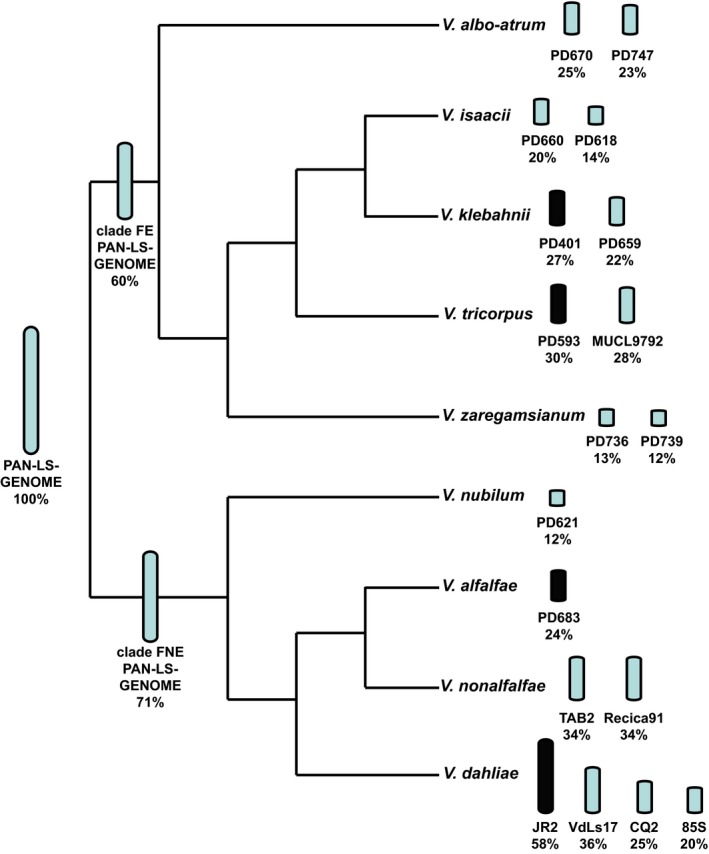
Diversity of pan‐LS‐genome contents across the *Verticillium* genus. A pan‐LS‐genome was constructed based on sequences from *V. dahliae* JR2, *V. alfalfae* PD683, *V. tricorpus* PD593 and *V. klebahnii* PD401 (black bars). The bar size next to the species names in the *Verticillium* phylogenetic tree is representative for the amount of the pan‐LS‐genome that is present in the individual isolates. All isolates of the clade Flavexudans (FE) in this study were used to calculate the percentage of the pan‐LS‐genome that is present in clade Flavexudans. Similarly, the portion of the Flavnonexudans (FNE) in the pan‐LS‐genome was calculated with all isolates of the clade Flavnonexudans used in this study [Colour figure can be viewed at wileyonlinelibrary.com]

Considering that horizontal transfer is unlikely, the high sequence identity between *Verticillium* LS sequences indicates that their origin is ancestral and predates speciation, and that lower numbers of DNA substitutions seem to occur in the LS regions. To determine the distribution of conserved LS sequences across the *Verticillium* genus, we constructed a pan‐LS‐genome by combining regions with high interspecific sequence identity of the four *Verticillium* species *V. dahliae* strain JR2, *V. alfalfae* strain PD683, *V. tricorpus* strain PD593, and *V. klebahnii* strain PD401 that were selected because of their high assembly contiguity and spread over the *Verticillium* genus (Inderbitzin et al., 2011; Shi‐Kunne et al., 2018). After removal of repetitive and duplicated sequences, we obtained a pan‐LS‐genome of ~2 Mb, of which 60% occurs in clade Flavexudans and 72% in clade Flavnonexudans (Figure 6). The proportion of the pan‐LS‐genome differed markedly between *Verticillium* strains and ranged from 12% for *V. nubilum* strain PD621 up to 58% for *V. dahliae* strain JR2 (Figure 6, Table S5). Notably, by using a limited number of isolates in the consensus reconstruction, retentions are probably biased towards strains that are phylogenetically closer related to the species that were used to compose the pan‐genome. However, *V. albo‐atrum* strains contained considerably more of the pan‐LS‐genome compared to *V. zaregamsianum* and *V. isaacii* strains, despite its phylogenetically more distant relation to *V. klebahnii* and *V. tricorpus* (Figure 6, Table S5). Moreover, LS contents do not only differ considerably between species but also within species. For example, the genome of *V. dahliae* strain VdLs17 contains less than two thirds of the content present in the JR2 genome despite the recent divergence of the two strains (Figures 1a and 4; Faino et al., 2015). Thus, sequences with high interspecific identity are associated with dynamic genomic regions of *Verticillium* species throughout the genus as their contents vary greatly between and within species.

### 3.6 Increased sequence conservation is probably not driven by negative selection

**Figure 7:**
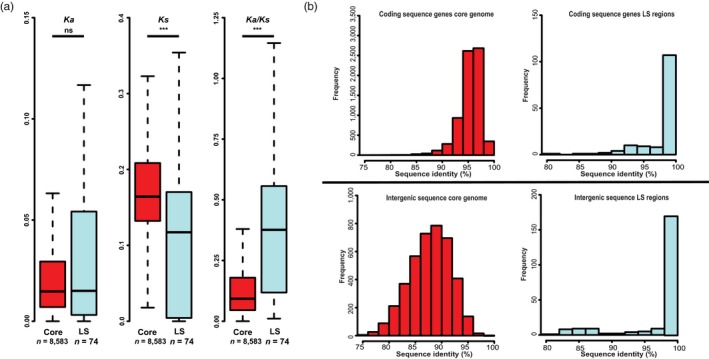
Comparison of substitutions of *Verticillium dahliae* strain JR2 and *V. nonalfalfae* strain TAB2 orthologs between core and lineage‐specific (LS) regions. (a) The distribution of non‐synonymous substitution rates (*Ka*), synonymous substitution rates (*Ks*) and *Ka*/*Ks* ratios are depicted for *V. dahliae* genes aligned to *V. nonalfalfae* orthologs. Outliers are not depicted. (b) Sequence identity of *V. dahliae* strain JR2 core and lineage‐specific (LS) regions with *V. nonalfalfae* strain TAB2 for coding and intergenic sequences. Coding sequences of *V. dahliae* strain JR2 genes were aligned to coding sequences of *V. nonalfalfae* strain TAB2 genes and the sequence identity was determined. For the intergenic regions, windows of 5 kb were constructed for *V. dahliae* strain JR2 core and LS regions. Significance of the different distributions was calculated with the two‐sided Wilcoxon rank‐sum test (ns, not significant; ***p < .001) [Colour figure can be viewed at wileyonlinelibrary.com]

To study the impact of the increased sequence conservation on gene evolution in more detail, substitution rates of LS region genes were compared with those of core region genes. We determined the rates of nonsynonymous (*Ka*) and synonymous (*Ks*) substitutions for LS versus core genes. In total, 48% (68 out of 142) of the LS genes could not be used for *Ka* and *Ks* determination, as we did not observe any substitutions when compared to their corresponding *V. nonalfalfae* orthologs. In contrast, almost all core genes (8,583 out of 8,584) display nucleotide substitutions when compared with their *V. nonalfalfae* orthologs. Whereas the *Ka* is not different (two‐sided Wilcoxon rank‐sum test, *p* < .05) between genes in LS regions (median = 0.015, *n* = 74) and the core genome (median = 0.015, *n* = 8,583), the *Ks* of genes in LS regions genes (median = 0.12, *n* = 74) is significantly lower than of core genes (median = 0.16, *n* = 8,583; Figure 7a). Consequently, LS genes (median = 0.38, *n* = 60) display significantly higher *Ka*/*Ks* values than core genes (median = 0.09, *n* = 8,289), calculated for genes that have both synonymous and nonsynonymous substitutions compared with their *V. nonalfalfae* orthologs. In total, 15 of the 74 tested LS genes displayed *Ka*/*Ks* > 1, which is a higher proportion than the 100 of the 8,583 core genes with *Ka*/*Ks* > 1 (Fisher's exact test, *p* < .05). Two LS and two core genes with *Ka*/*Ks* > 1 were predicted to contain an N‐terminal signal peptide, which is a typical characteristic of effector proteins. However, due to the limited sequence divergence in the LS regions, positive selection on the genes with *Ka*/*Ks* > 1 was not significant based on a *Z* test, whereas in the core genome 21 genes were found to be under positive selection (*p* < .05). Thus, a similar fraction of nucleotide alterations originates from nonsynonymous substitutions in LS and core genome regions, whereas synonymous substitutions occur less frequent in LS regions when compared with the core genome. The resulting increase in *Ka*/*Ks* ratio's for genes in LS regions suggests that negative selection is probably not responsible for the slower divergence of gene sequences. Rather, LS regions seem to encounter a lower rate of synonymous nucleotide substitutions that lead to lower numbers of neutral/synonymous substitutions in genes in LS regions when compared with core genes. In congruence with this hypothesis, sequence comparisons of coding and intergenic regions between *V. dahliae* and *V. nonalfalfae* revealed that increased sequence conservation similarly occurs in coding regions as well as intergenic sequences (Figure 7b), suggesting that the increased sequence conservation is driven by a mechanism that affects whole LS regions, rather than by selection.

## 4 DISCUSSION

Genomes of many filamentous plant pathogens are thought to obey to a two‐speed evolution model (Croll & McDonald, 2012; Dong et al., 2015; Möller & Stukenbrock, 2017). *Verticillium dahliae* is similarly thought to evolve under a two‐speed regime, as LS regions that are of significance for host interactions display increased structural variation and TE activity (Faino et al., 2015, 2016; de Jonge et al., 2013). Additionally, LS regions are extremely plastic with abundant presence/absence polymorphisms (Figure 1; Faino et al., 2016; de Jonge et al., 2013). Intriguingly, although LS regions are enriched in segmental duplications (Faino et al., 2016), which can be an incentive for evolutionary diversification (Magadum et al., 2013), LS sequences display a remarkably high degree of sequence identity to other *Verticillium* species (Figures 2, 3; Table 1). Principally, sequences with increased identities between distinct taxa can originate from horizontal transfer, a phenomenon that has been implicated in the pathogenicity of various filamentous plant pathogens (Soanes & Richards, 2014). For instance, *Pyrenophora tritici‐repentis*, the causal agent of wheat tan spot, acquired a gene from the fungal wheat pathogen *Phaeosphaeria nodorum* enabling the production of the host‐specific toxin ToxA that mediates pathogenicity on wheat (Friesen et al., 2006). However, horizontal transfer is not likely to explain our observations, as the increased sequence identity is observed genus‐wide and concerns every species within the genus (Table 1). If the increased sequence conservation would be caused by horizontal transfers, *V. dahliae* and *V. tricorpus* each should have been involved in at least five such transfers during their evolution to explain the difference in median sequence identity between LS and core genome regions (Figure 4, Table 1). Thus, by only considering these two species at least nine interspecific DNA transfers must have occurred during the evolution of the *Verticillium* genus. Phylogenetic trees of LS genes suggest that the majority of genes evolved according the *Verticillium* clade Flavnonexudans and Flavexudans dichotomy, indicating that most genes do not originate from horizontal transfer between these two phylogenetic clades. Furthermore, individual cases of LS genes for which (near‐)identical orthologs can be found in up to six of the nine haploid *Verticillium* species (Figure 5) further argue against horizontal transfer, especially when considering the low‐mobile, soil‐borne nature of the various species.

Considering that horizontal transfer is extremely unlikely, we argue that *V. dahliae* LS regions are subject to processes that mediate increased sequence conservation. This increased sequence conservation of LS regions would not be a consequence of negative selection on coding regions, as intergenic regions display similarly increased conservation levels (Figure 7b). Moreover, LS genes display a similar fraction of nonsynonymous substitutions (*Ka*) as core genes, but carry significantly less synonymous substitutions (*Ks*; Figure 7a). Consequently, high interspecific sequence identity of LS genome regions is probably due to generally lower synonymous nucleotide substitution rates in these regions when compared with the core genome.

Repressed levels of synonymous substitutions were previously found for repeat‐rich dispensable chromosomes of the fungal wheat pathogen *Zymoseptoria tritici* (Stukenbrock et al., 2010). However, this observation was not attributed to lower substitution rates, but rather the consequence of a lower effective population size of these dispensable chromosomes (Stukenbrock et al., 2010). As sex is probably rare to nonexistent in *Verticillium*, selective sweeps are an unlikely explanation for increased sequence conservation of LS region as, in case of *Verticillium* species with low to nonexistent meiotic recombination, entire genomes would sweep to fixation and not specific genome regions (Shapiro, Leducq, & Mallet, 2016). Moreover, sequence conservation is observed between species and fixation of the same genome region across species boundaries is unlikely, as different *Verticillium* species conceivably encounter distinct selection pressures due to differences in their biological features, such as pathogenicity and host ranges (Inderbitzin & Subbarao, 2014). Thus, the increased sequence conservation as observed here is unprecedented and perhaps counter‐intuitive. Previously, increased substitution rates have been associated with two‐speed genome evolution (Cuomo et al., 2007; Dong et al., 2015). For example, repeat‐induced point (RIP) mutagenesis increases sequence divergence of particular effector genes of the oilseed rape pathogen *Leptosphaeria maculans* that are localized near TEs (van de Wouw et al., 2010). However, accelerated evolution through increased SNP frequencies is not consistently observed for two‐speed genomes, as no significant difference in SNP frequencies between core and repeat‐rich genomic regions was found in *P. infestans* (Raffaele et al., 2010). Nevertheless, accelerated evolution of LS regions can also be established through other means, such as presence/absence polymorphisms. The well‐characterized *V. dahliae* LS effector *Ave1* is highly conserved, as an identical copy occurs *V. alfalfae* strain VaMs102 that displays a genome‐wide average sequence identity of 92% (de Jonge et al., 2012). Moreover, no *Ave1* allelic variation is hitherto found in the *V. dahliae* population as well as in *V. alfalfae* and *V. nonalfalfae* populations (de Jonge et al., 2012; Song et al., 2017). Since Ave1 is recognized by the tomato immune receptor Ve1 (Fradin et al.., 2009), evasion of recognition occurs through various *Ave1* deletion events from the population (Faino et al., 2016; de Jonge et al., 2012).

Mechanisms that can explain the observed increased sequence conservation in repeat‐rich LS regions remain unknown. Mutations mostly originate from DNA polymerase errors and there is no immediate reason why these error rates would diverge in LS regions. Possibly, the depletion of nucleotide substitutions can be associated with a differential chromatin organisation of LS regions. Intriguingly, a study into chromatin structure in the human genome noted that regions of open chromatin displayed lower mutation rates which was hypothesized to be a result of these regions being more accessible to repair mechanisms (Prendergast et al., 2007). However, repeat‐rich regions such as the LS regions in *V. dahliae* are thought to be associated with densely organised chromatin, referred to as heterochromatin (Galazka & Freitag, 2014). In *Z. tritici*, repeat‐rich conditionally dispensable chromosomes are enriched for histone modifications associated with heterochromatin, in contrast to core chromosomes that are largely euchromatic and transcriptionally active (Schotanus et al., 2015). Generally, heterochromatin is associated with suppression of genomic structural alterations such as recombination. Nevertheless, heterochromatic regions of *Z. tritici* are enriched for structural variations as they are enriched for duplications and deletions (Seidl, Cook, & Thomma, 2016). Thus, further research is needed to investigate whether differences in chromatin organisation can explain lower rates of SNP frequencies that occur in the plastic LS regions of *V. dahliae*.

## 5 CONCLUSION

The two‐speed genome is an intuitive evolutionary model for filamentous pathogens, as genes important for pathogenicity benefit from frequent alternations to mediate continued symbiosis with the host. However, filamentous pathogens comprise a heterogeneous group of organisms with diverse lifestyles (Dean et al., 2012; Kamoun et al., 2015). Consequently, it is not surprising that accelerated evolution is driven by different mechanisms between species. In *V. dahliae*, acceleration evolution is merely achieved through presence/absence polymorphisms, as nucleotide sequences are highly conserved in LS regions. Perhaps, deletion of recognized effectors leads to a more rapid immunity evasion than sequence alterations through nucleotide substitutions (Daverdin et al., 2012). Thus, the quick fashion of host immunity evasion through the deletion of effector genes can be evolutionary advantageous over allelic diversification, especially for soil‐borne pathogens with a small effective population size that have little means of mobility.

## AUTHOR CONTRIBUTIONS

X.S.K., H.M., T.L., L.F., G.C.M.B., B.Z., and A.J. contributed to the data generation of this study. J.R.L.D. performed the comparative genomic analysis and wrote the manuscript with the help of X.S.K., M.F.S., and B.P.H.J.T., J.R.L.D., X.S.K., M.F.S., and B.P.H.J.T. contributed to the study design. T.W., M.F.S., and B.P.H.J.T. supervised this study.

## Data Availability

The Whole Genome Shotgun projects have been deposited at DDBJ/ENA/GenBank as accessions PRLI00000000 and PRLJ00000000 for *V. dahliae* strains CQ2 and 85S, respectively.

## References

[mec15168-bib-0001] Altschul, S. F. , Gish, W. , Miller, W. , Myers, E. W. , & Lipman, D. J. (1990). Basic local alignment search tool. Journal of Molecular Biology, 215, 403–410. 10.1016/S0022-2836(05)80360-2 2231712

[mec15168-bib-0002] Bertels, F. , Silander, O. K. , Pachkov, M. , Rainey, P. B. , & Van Nimwegen, E. (2014). Automated reconstruction of whole‐genome phylogenies from short‐sequence reads. Molecular Biology and Evolution, 31, 1077–1088. 10.1093/molbev/msu088 24600054 PMC3995342

[mec15168-bib-0003] Chin, C.‐S. , Alexander, D. H. , Marks, P. , Klammer, A. A. , Drake, J. , Heiner, C. , … Korlach, J. (2013). Nonhybrid, finished microbial genome assemblies from long‐read SMRT sequencing data. Nature Methods, 10, 563–569. 10.1038/nmeth.2474 23644548

[mec15168-bib-0004] Cook, D. E. , Mesarich, C. H. , & Thomma, B. P. H. J. (2015). Understanding plant immunity as a surveillance system to detect invasion. Annual Review of Phytopathology, 53, 541–563. 10.1146/annurev-phyto-080614-120114 26047564

[mec15168-bib-0005] Croll, D. , & McDonald, B. A. (2012). The accessory genome as a cradle for adaptive evolution in pathogens. PLoS Pathogens, 8, e1002608 10.1371/journal.ppat.1002608 22570606 PMC3343108

[mec15168-bib-0006] Cuomo, C. A. , Guldener, U. , Xu, J.‐R. , Trail, F. , Turgeon, B. G. , Di Pietro, A. , … Kistler, H. C. (2007). The *Fusarium graminearum* genome reveals a link between localized polymorphism and pathogen specialization. Science, 317, 1400–1402. 10.1126/science.1143708 17823352

[mec15168-bib-0007] Daverdin, G. , Rouxel, T. , Gout, L. , Aubertot, J.‐N. , Fudal, I. , Meyer, M. , … Balesdent, M.‐H. (2012). Genome structure and reproductive behaviour influence the evolutionary potential of a fungal phytopathogen. PLoS Pathogens, 8, e1003020 10.1371/journal.ppat.1003020 23144620 PMC3493481

[mec15168-bib-0008] de Jonge, R. , Bolton, M. D. , Kombrink, A. , van den Berg, G. C. , Yadeta, K. A. , & Thomma, B. P. (2013). Extensive chromosomal reshuffling drives evolution of virulence in an asexual pathogen. Genome Research, 23, 1271–1282. 10.1101/gr.152660.112 23685541 PMC3730101

[mec15168-bib-0009] de Jonge, R. , Peter van Esse, H. , Maruthachalam, K. , Bolton, M. D. , Santhanam, P. , Saber, M. K. , … Thomma, B. P. H. J. (2012). Tomato immune receptor Ve1 recognizes effector of multiple fungal pathogens uncovered by genome and RNA sequencing. Proceedings of the National Academy of Sciences of the USA, 109, 5110–5115. 10.1073/pnas.1119623109 22416119 PMC3323992

[mec15168-bib-0010] Dean, R. , van Kan, J. A. L. , Pretorius, Z. A. , Hammond‐kosack, K. E. , Di pietro, A. , Spanu, P. D. , … Foster, G. D. (2012). The top 10 fungal pathogens in molecular plant pathology. Molecular Plant Pathology, 13, 414–430. 10.1111/j.1364-3703.2011.00783.x 22471698 PMC6638784

[mec15168-bib-0011] Dodds, P. N. , & Rathjen, J. P. (2010). Plant immunity: Towards an integrated view of plant–pathogen interactions. Nature Reviews Genetics, 11, 539–548. 10.1038/nrg2812 20585331

[mec15168-bib-0012] Dong, S. , Raffaele, S. , & Kamoun, S. (2015). The two‐speed genomes of filamentous pathogens: Waltz with plants. Current Opinion in Genetics & Development, 35, 57–65. 10.1016/j.gde.2015.09.001 26451981

[mec15168-bib-0013] Emms, D. M. , & Kelly, S. (2015). OrthoFinder: Solving fundamental biases in whole genome comparisons dramatically improves orthogroup inference accuracy. Genome Biology, 6, 157 10.1186/s13059-015-0721-2 PMC4531804 26243257

[mec15168-bib-0014] Faino, L. , Seidl, M. , Datema, E. , van den Berg, G. C. M. , Janssen, A. , Wittenberg, A. H. J. , … Thomma, B. P. H. J. (2015). Single‐molecule real‐time sequencing combined with optical mapping yields completely finished fungal genome. MBio, 6, e00936‐15 10.1128/mBio.00936-15 26286689 PMC4542186

[mec15168-bib-0015] Faino, L. , Seidl, M. F. , Shi‐Kunne, X. , Pauper, M. , van den Berg, G. C. M. , Wittenberg, A. H. J. , & Thomma, B. P. H. J. (2016). Transposons passively and actively contribute to evolution of the two‐speed genome of a fungal pathogen. Genome Research, 26, 1091–1100. 10.1101/gr.204974.116 27325116 PMC4971763

[mec15168-bib-0016] Fradin, E. F. , & Thomma, B. P. H. J. (2006). Physiology and molecular aspects of Verticillium wilt diseases caused by *V. dahliae* and *V. albo‐atrum* . Molecular Plant Pathology, 7, 71–86. 10.1111/j.1364-3703.2006.00323.x 20507429

[mec15168-bib-0017] Fradin, E. F. , Zhang, Z. , Juarez Ayala, J. C. , Castroverde, C. D. , Nazar, R. N. , Robb, J. , … Thomma, B. P. (2009). Genetic dissection of Verticillium wilt resistance mediated by tomato Ve1. Plant Physiology, 150, 320–332. 19321708 10.1104/pp.109.136762 PMC2675724

[mec15168-bib-0018] Friesen, T. L. , Stukenbrock, E. H. , Liu, Z. , Meinhardt, S. , Ling, H. , Faris, J. D. , … Oliver, R. P. (2006). Emergence of a new disease as a result of interspecific virulence gene transfer. Nature Genetics, 38, 953–956. 10.1038/ng1839 16832356

[mec15168-bib-0019] Galazka, J. M. , & Freitag, M. (2014). Variability of chromosome structure in pathogenic fungi‐of ‘ends and odds’. Current Opinion in Microbiology, 20, 19–26. 10.1016/j.mib.2014.04.002 24835423 PMC4133287

[mec15168-bib-0020] Grum‐Grzhimaylo, A. A. , Falkoski, D. L. , van den Heuvel, J. , Valero‐Jiménez, C. A. , Min, B. , Choi, I. G. , … Debets, A. J. M. (2018). The obligate alkalophilic soda‐lake fungus *Sodiomyces alkalinus* has shifted to a protein diet. Molecular Ecology, 27, 4808–4819. 30368956 10.1111/mec.14912

[mec15168-bib-0021] Gurevich, A. , Saveliev, V. , Vyahhi, N. , & Tesler, G. (2013). QUAST: Auality assessment tool for genome assemblies. Bioinformatics, 29, 1072–1075. 23422339 10.1093/bioinformatics/btt086 PMC3624806

[mec15168-bib-0022] Guy, L. , Kultima, J. R. , & Andersson, S. G. E. (2011). GenoPlotR: Comparative gene and genome visualization in R. Bioinformatics, 26, 2334–2335. 10.1093/bioinformatics/btq413 PMC2935412 20624783

[mec15168-bib-0023] Haas, B. J. , Kamoun, S. , Zody, M. C. , Jiang, R. H. Y. , Handsaker, R. E. , Cano, L. M. , … Nusbaum, C. (2009). Genome sequence and analysis of the Irish potato famine pathogen *Phytophthora infestans* . Nature, 461, 393–398. 10.1038/nature08358 19741609

[mec15168-bib-0024] Holt, C. , & Yandell, M. (2011). MAKER2: An annotation pipeline and genome‐ database management tool for second‐ generation genome projects. BMC Bioinformatics, 12, 491 10.1186/1471-2105-12-491 22192575 PMC3280279

[mec15168-bib-0025] Inderbitzin, P. , Bostock, R. M. , Davis, R. M. , Usami, T. , Platt, H. W. , & Subbarao, K. V. (2011). Phylogenetics and taxonomy of the fungal vascular wilt pathogen *Verticillium*, with the descriptions of five new species. PLoS ONE, 6, e28341 10.1371/journal.pone.0028341 22174791 PMC3233568

[mec15168-bib-0026] Inderbitzin, P. , & Subbarao, K. V. (2014). *Verticillium* systematics and evolution: How confusion impedes Verticillium wilt management and how to resolve it. Phytopathology, 104, 564–574. 24548214 10.1094/PHYTO-11-13-0315-IA

[mec15168-bib-0027] Jelen, V. , de Jonge, R. , Van de Peer, Y. , Javornik, B. , & Jakse, J. (2016). Complete mitochondrial genome of the Verticillium‐wilt causing plant pathogen *Verticillium nonalfalfae* . PLoS ONE, 11, e0148525 10.1371/journal.pone.0148525 26839950 PMC4739603

[mec15168-bib-0028] Kamoun, S. , Furzer, O. , Jones, J. D. G. , Judelson, H. S. , Ali, G. S. , Dalio, R. J. D. , … Govers, F. (2015). The Top 10 oomycete pathogens in molecular plant pathology. Molecular Plant Pathology, 16, 413–434. 10.1111/mpp.12190 25178392 PMC6638381

[mec15168-bib-0029] Katoh, K. , Misawa, K. , Kuma, K. , & Miyata, T. (2002). MAFFT: A novel method for rapid multiple sequence alignment based on fast Fourier transform. Nucleic Acids Research, 30, 3059–3066. 10.1093/nar/gkf436 12136088 PMC135756

[mec15168-bib-0030] Katoh, K. , & Standley, D. M. (2013). MAFFT multiple sequence alignment software version 7: Improvements in performance and usability. Molecular Biology and Evolution, 30, 772–780. 10.1093/molbev/mst010 23329690 PMC3603318

[mec15168-bib-0031] Klosterman, S. J. , Subbarao, K. V. , Kang, S. , Veronese, P. , Gold, S. E. , Thomma, B. P. H. J. , … Ma, L.‐J. (2011). Comparative genomics yields insights into niche adaptation of plant vascular wilt pathogens. PLoS Pathogens, 7, e1002137 10.1371/journal.ppat.1002137 21829347 PMC3145793

[mec15168-bib-0032] Kurtz, S. , Phillippy, A. , Delcher, A. L. , Smoot, M. , Shumway, M. , Antonescu, C. , & Salzberg, S. L. (2004). Versatile and open software for comparing large genomes. Genome Biology, 5, R12. 14759262 10.1186/gb-2004-5-2-r12 PMC395750

[mec15168-bib-0033] Luo, R. , Liu, B. , Xie, Y. , Li, Z. , Huang, W. , Yuan, J. , … Wang, J. (2012). SOAPdenovo2: An empirically improved memory‐efficient short‐read de novo assembler. GigaScience, 1, 18 10.1186/2047-217X-1-18 23587118 PMC3626529

[mec15168-bib-0034] Magadum, S. , Banerjee, U. , Murugan, P. , Gangapur, D. , & Ravikesavan, R. (2013). Gene duplication as a major force in evolution. Journal of Genetics, 92, 155–161. 10.1007/s12041-013-0212-8 23640422

[mec15168-bib-0035] Möller, M. , & Stukenbrock, E. H. (2017). Evolution and genome architecture in fungal plant pathogens. Nature Reviews Microbiology, 15, 756–771. 10.1038/nrmicro.2017.76 29123226

[mec15168-bib-0036] Nei, M. , & Gojobori, T. (1986). Simple methods for estimating the numbers of synonymous and nonsynonymous nucleotide substitutions. Molecular Biology and Evolution, 3, 418–426. 3444411 10.1093/oxfordjournals.molbev.a040410

[mec15168-bib-0037] Petersen, T. N. , Brunak, S. , Von Heijne, G. , & Nielsen, H. (2011). SignalP 4.0: Discriminating signal peptides from transmembrane regions. Nature Methods, 8, 785–786. 10.1038/nmeth.1701 21959131

[mec15168-bib-0038] Pikuta, E. V. , Hoover, R. B. , & Tang, J. (2007). Microbial extremophiles at the limits of life. Critical Reviews in Microbiology, 33, 183–209. 10.1080/10408410701451948 17653987

[mec15168-bib-0039] Prendergast, J. G. D. , Campbell, H. , Gilbert, N. , Dunlop, M. G. , Bickmore, W. A. , & Semple, C. A. M. (2007). Chromatin structure and evolution in the human genome. BMC Evolutionary Biology, 7, 72 10.1186/1471-2148-7-72 17490477 PMC1876461

[mec15168-bib-0040] Quinlan, A. R. , & Hall, I. M. (2010). BEDTools: A flexible suite of utilities for comparing genomic features. Bioinformatics, 26, 841–842. 10.1093/bioinformatics/btq033 20110278 PMC2832824

[mec15168-bib-0041] Raffaele, S. , Farrer, R. A. , Cano, L. M. , Studholme, D. J. , MacLean, D. , Thines, M. , … Kamoun, S. (2010). Genome evolution following host jumps in the Irish potato famine pathogen lineage. Science, 330, 1540–1543. 10.1126/science.1193070 21148391

[mec15168-bib-0042] Raffaele, S. , & Kamoun, S. (2012). Genome evolution in filamentous plant pathogens: Why bigger can be better. Nature Reviews Microbiology, 10, 417–430. 10.1038/nrmicro2790 22565130

[mec15168-bib-0043] Schotanus, K. , Soyer, J. L. , Connolly, L. R. , Grandaubert, J. , Happel, P. , Smith, K. M. , … Stukenbrock, E. H. (2015). Histone modifications rather than the novel regional centromeres of *Zymoseptoria tritici* distinguish core and accessory chromosomes. Epigenetics & Chromatin, 8, 41 10.1186/s13072-015-0033-5 26430472 PMC4589918

[mec15168-bib-0044] Seidl, M. F. , Cook, D. E. , & Thomma, B. P. H. J. (2016). Chromatin biology impacts adaptive evolution of filamentous plant pathogens. PLoS Pathogens, 12, e1005920 10.1371/journal.ppat.1005920 27812218 PMC5094656

[mec15168-bib-0045] Seidl, M. F. , Faino, L. , Shi‐Kunne, X. , van den Berg, G. C. , Bolton, M. D. , & Thomma, B. P. (2015). The genome of the saprophytic fungus *Verticillium tricorpus* reveals a complex effector repertoire resembling that of its pathogenic relatives. Molecular Plant‐Microbe Interactions, 28, 362–373. 25208342 10.1094/MPMI-06-14-0173-R

[mec15168-bib-0046] Seidl, M. F. , & Thomma, B. P. H. J. (2014). Sex or no sex: Evolutionary adaptation occurs regardless. BioEssays, 36, 335–345. 10.1002/bies.201300155 24531982 PMC4158867

[mec15168-bib-0047] Shapiro, B. J. , Leducq, J.‐B. , & Mallet, J. (2016). What is speciation? PLoS Genetics, 12, e1005860 10.1371/journal.pgen.1005860 27030977 PMC4816541

[mec15168-bib-0048] Shi‐Kunne, X. , Faino, L. , van den Berg, G. C. M. , Thomma, B. P. H. J. , & Seidl, M. F. (2018). Evolution within the fungal genus *Verticillium* is characterized by chromosomal rearrangements and gene losses. Environmental Microbiology, 20, 1362–1373. 29282842 10.1111/1462-2920.14037

[mec15168-bib-0049] Shi‐Kunne, X. , van Kooten, M. , Depotter, J. R. L. , Thomma, B. P. H. J. , & Seidl, M. F. (2019). The genome of the fungal pathogen *Verticillium dahliae* reveals extensive bacterial to fungal gene transfer. Genome Biology and Evolution, 11, 855–868. 30799497 10.1093/gbe/evz040 PMC6430987

[mec15168-bib-0050] Short, D. P. G. , Gurung, S. , Hu, X. , Inderbitzin, P. , & Subbarao, K. V. (2014). Maintenance of sex‐related genes and the co‐occurrence of both mating types in *Verticillium dahliae* . PLoS ONE, 9, e112145 10.1371/journal.pone.0112145 25383550 PMC4226480

[mec15168-bib-0051] Smit, A. F. A. , Hubley, R. , & Green, P. (2015). RepeatMasker Open‐4.0. Retrieved from http://www.repeatmasker.org

[mec15168-bib-0052] Soanes, D. , & Richards, T. A. (2014). Horizontal gene transfer in eukaryotic plant pathogens. Annual Review of Phytopathology, 52, 583–614. 10.1146/annurev-phyto-102313-050127 25090479

[mec15168-bib-0053] Song, Y. , Zhang, Z. , Boshoven, J. C. , Rovenich, H. , Seidl, M. F. , Jakše, J. , … Thomma, B. P. H. J. (2017). Tomato immune receptor Ve1 recognizes surface‐exposed co‐localized N‐ and C‐termini of *Verticillium dahliae* effector Ave1. BioRxiv. 10.1101/103473

[mec15168-bib-0054] Sperschneider, J. , Gardiner, D. M. , Thatcher, L. F. , Lyons, R. , Singh, K. B. , Manners, J. M. , & Taylor, J. M. (2015). Genome‐wide analysis in three *Fusarium* pathogens identifies rapidly evolving chromosomes and genes associated with pathogenicity. Genome Biology and Evolution, 7, 1613–1627. 10.1093/gbe/evv092 25994930 PMC4494044

[mec15168-bib-0055] Stamatakis, A. (2014). RAxML version 8: A tool for phylogenetic analysis and post‐analysis of large phylogenies. Bioinformatics, 30, 1312–1313. 10.1093/bioinformatics/btu033 24451623 PMC3998144

[mec15168-bib-0056] Stukenbrock, E. H. , & Dutheil, J. Y. (2012). Comparing fungal genomes: Insight into functional and evolutionary processes. Methods in Molecular Biology, 835, 531–548. 22183676 10.1007/978-1-61779-501-5_33

[mec15168-bib-0057] Stukenbrock, E. H. , Jørgensen, F. G. , Zala, M. , Hansen, T. T. , McDonald, B. A. , & Schierup, M. H. (2010). Whole‐genome and chromosome evolution associated with host adaptation and speciation of the wheat pathogen *Mycosphaerella graminicola* . PLoS Genetics, 6, e1001189 10.1371/journal.pgen.1001189 21203495 PMC3009667

[mec15168-bib-0058] Tritt, A. , Eisen, J. A. , Facciotti, M. T. , & Darling, A. E. (2012). An integrated pipeline for de novo assembly of microbial genomes. PLoS ONE, 7, e42304 10.1371/journal.pone.0042304 23028432 PMC3441570

[mec15168-bib-0059] van de Wouw, A. P. , Cozijnsen, A. J. , Hane, J. K. , Brunner, B. A. , McDonald, B. A. , Oliver, R. P. , & Howlett, B. J. (2010). Evolution of linked avirulence effectors in *Leptosphaeria maculans* is affected by genomic environment and exposure to resistance genes in host plants. PLoS Pathogens, 6, e1001180 10.1371/journal.ppat.1001180 21079787 PMC2973834

[mec15168-bib-0060] Yang, Z. (2007). PAML 4: Phylogenetic analysis by maximum likelihood. Molecular Biology and Evolution, 24, 1586–1591. 10.1093/molbev/msm088 17483113

[mec15168-bib-0061] Zhang, H. , Meltzer, P. , & Davis, S. (2013). RCircos: An R package for Circos 2D track plots. BMC Bioinformatics, 14, 244 10.1186/1471-2105-14-244 23937229 PMC3765848

