# Supplementary material for: Dynamic virulence‐related regions of the plant pathogenic fungus Verticillium dahliae display enhanced sequence conservation
Source: Mol Ecol. 2019 Jul 29;28(15):3482–95. doi: 10.1111/mec.15168 (PMC6771948; doi:10.1111/mec.15168)
Supplement: Supplementary file 1 [file MEC-28-3482-s001.pdf]

SUPPORTING INFORMATION

Dynamic virulence-related regions of the plant pathogenic fungus *Verticillium dahliae* display enhanced sequence conservation

Jasper R.L. Depotter, Xiaoqian Shi-Kunne, Hélène Missonnier, Tingli Liu, Luigi Faino, Grardy C.M. van den Berg, Thomas A. Wood, Baolong Zhang, Alban Jacques, Michael F. Seidl, Bart P.H.J. Thomma

Table S1: Genome assemblies of *Verticillium dahliae* strains CQ2 and 85S.

		**CQ2**	**85S**
SMRT cells		4	4
Filtered subreads		430,378	500,428
Coverage (n-fold)		110x	130x
HGAP3 assembly	Size (bp)	35,818,019	35,931,336
	Contigs	17	40
	Longest contig	7,864,170	6,633,769
	N_{50} (bp)	3,754,186	3,176,090
	No. of Ns/100 kb	0	0

Table S2. Assembly statistics of the *Verticillium* genomes used in this study.

Species	Strain name	Genome size (Mb)	#Ns/100 kb	N50 (Mb)	# Contigs (≥0 bp)	# Scaffolds (≥1000 bp)
V. albo-atrum	PD670	37.4	56.67	3.7	107	72
	PD747	36.5	16.26	3.9	34	19
V. alfalfae	PD683	32.7	19.36	4.5	40	14
V. dahliae	VdLs17	36.0	0	5.9	8	8
	JR2	36.2	0	4.2	8	8
	CQ2	35.8	0	3.8	17	17
	85S	35.9	0	3.2	40	40
V. nonalfalfae	Rec	33.0	35.4	0.9	1026	795
	TAB2	34.3	897.53	1.8	793	167
V. nubilum	PD621	37.9	9.13	4.7	246	189
V. tricorpus	MUCL9792	36.0	229,64	4,7	255	53

	PD593	35.0	14.52	4.4	71	9
V. isaacii	PD618	35.8	62.48	3.1	239	122
	PD660	36.0	37.53	2.5	114	43
V. klebahnii	PD659	36.2	59.47	3.6	120	60
	PD401	36.0	35.30	3.2	79	37
V. zaregamsianum	PD736	37.1	62.7	2.0	125	62
	PD739	37.1	55.38	3.5	75	32

Table S3. Sequences of other haploid *Verticillium* species with high identity to *Verticillium dahliae* strain JR2.

Species	Strain name	Amount of high identity sequences (kb)	Fraction that align to LS regions (%)
V. albo-atrum[‡]	PD670	209	94.6
	PD747	160	91.9
V. alfalfae[†]	PD683	473	99.5
V. nonalfalfae[†]	Rec	662	100.0
	TAB2	705	99.9
V. nubilum[‡]	PD621	147	69.6
V. tricorpus[‡]	MUCL9792	92	76.1
	PD593	304	95.3
V. isaacii[‡]	PD618	153	89.5
	PD660	202	89.9
V. klebahnii[‡]	PD659	193	86.6
	PD401	142	81.9
V. zaregamsianum[‡]	PD736	54	52.4
	PD739	68	86.8

† Sequences with an identity of >95% with *V. dahliae* JR2
‡ Sequences with an identity of >90% with *V. dahliae* JR2

Table S4. Sequences of other haploid *Verticillium* species with high identity to *Verticillium tricorpus* strain PD593.

Species	Strain name	Amount of high identity sequences (kb)	Fraction that align to the two regions with high sequence identity (%)
V. albo-atrum[‡]	PD670	510	83.4
	PD747	527	83.8
V. alfalfae[‡]	PD683	46	1.3
V. dahliae[‡]	VdLs17	153	80.6
	JR2	285	81.1
	CQ2	36	3.1
	85S	67	2.1
V. nonalfalfae[‡]	Rec	322	70.0
	TAB2	60	30.0
V. nubilum[‡]	PD621	133	49.4
V. isaacii[†]	PD618	63	32.6
	PD660	100	60.4
V. klebahnii[†]	PD659	105	62.5
	PD401	49	18.8
V. zaregamsianum[†]	PD736	44	9.8
	PD739	40	7.3

† Sequences with an identity of >95% with *V. tricorpus* PD593

‡ Sequences with an identity of >90% with *V. tricorpus* PD593

Table S5: Presence of constructed dynamic regions in individual *Verticillium* strains.

**Species**	**Strain**	**Consensus**	**Clade FE[†] consensus**	**Clade FNE[‡] consensus**
V. albo-atrum	PD670	25%	41%	21%
	PD747	23%	38%	17%
V. alfalfae	PD683	24%	9%	34%
V. dahliae	VdLs17	36%	27%	51%
	JR2	58%	39%	81%
	CQ2	25%	12%	35%
	85S	20%	8%	28%
V. nonalfalfae	Rec	34%	28%	48%
	TAB2	34%	21%	47%
V. nubilum	PD621	12%	16%	17%
V. tricorpus	MUCL9792	28%	48%	15%
	PD593	30%	50%	19%
V. isaacii	PD618	14%	24%	15%
	PD660	20%	34%	17%
V. klebahnii	PD659	22%	38%	16%
	PD401	27%	46%	14%
V. zaregamsianum	PD736	13%	21%	11%
	PD739	12%	20%	12%

†: FE = Flavexudans; ‡: FNE = Flavnonexudans

Table S6: Genome regions used for pan-LS-genome construction.

Species (strain)	Chromosome/scaffold	Start and end position
V. dahlia (JR2)	Chr2	2871432-3666484
	Chr4	937294-1480748
	Chr5	560178-1061663
	Chr5	3387969-3743452
V. alfalfa (PD683)	Sca11	1-171020
	Sca12	1-105165
	Sca13	1-98982
V. tricorpus (PD589)	Sca1	971738-1381898
	Sca6	3245714-3479666
V. klebahnii (PD401)	Sca6	1566798-1795658
	Sca21	200168-272356
	Sca22	1-101836

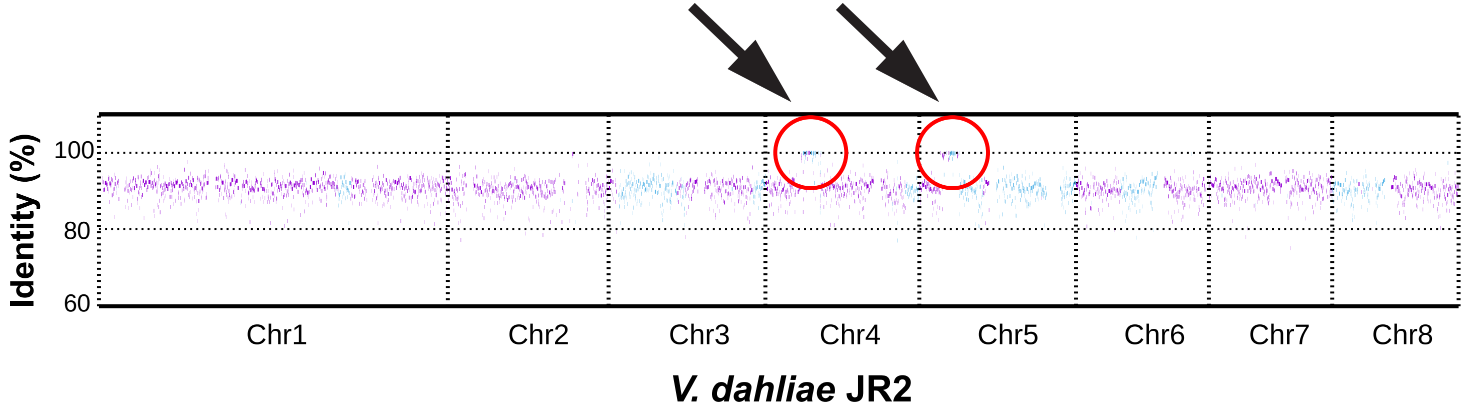

Figure S1: Whole-genome coverage plot displaying identity of aligned sequences of *V. nonalfalfae* strain TAB2 to *V. dahliae* strain JR2. Aligned genomic regions are displayed and purple and blue colours indicate forward-forward alignments and forward-reverse alignments (inversions), respectively. Genome regions with black arrows indicate 99-100% sequence identity.
